# Antioxidant activity, acetylcholinesterase inhibitory potential and phytochemical analysis of *Sarcocephalus latifolius* Sm. bark used in traditional medicine in Sudan

**DOI:** 10.1186/s12906-017-1772-6

**Published:** 2017-05-18

**Authors:** Alsiddig Osama, Sufyan Awadelkarim, Amna Ali

**Affiliations:** 1 Chemistry Department, Omdurman Islamic University, P.O. Box 382, Omdurman, Sudan; 2 Medical Biochemistry Research Unit, Medicinal and Aromatic Plants Research Institute, National Centre for Research, P.O. Box 2404, Khartoum, Sudan

**Keywords:** *S. latifolius*, Antioxidant, Acetylcholinesterase, Phenolics, Flavonoids, Gcms

## Abstract

**Background:**

*Sarcocephalus latifolius* is used as a traditional medicine for curing many diseases in Sudan. The main objective of the current study was to determine the antioxidant activity and acetylcholinesterase inhibition (AChEI) of *S. latifolius*, and to estimate its total phenolic and flavonoid contents.

**Methods:**

Antioxidant activity of the tested plant extracts was carried out by determining their ability to scavenge the 2,2-diphenyl-1-picryl hydrazyl (DPPH) free radical. On the other hand, AChE inhibitory activity was determined spectrophotometrically using the Ellman’s colorimetric method. The levels of total phenols and flavonoids were determined quantitatively using spectrophotometric methods. MTT assay was consumed to assess the cytotoxic effect of the most active fractions. These fractions were subjected to phytochemical analysis using GC-MS techniques to determine thier chemical composition.

**Results:**

Hexane and chloroform fractions exhibited the highest antioxidant activity with IC_50_ values of (0.098 ± 0.08 and 0.099 ± 0.029 mg/ml) respectively. Standard propyl gallate had the lowest IC_50_ value of 0.0414 ± 0.11 mg/ml. The ethanolic crude extract showed low AChEI activity with 40.2 ± 0.10%. High concentrations of phenolic and flavonoid contents were observed. GCMS revealed the presence of well-known antioxidants compounds e.g. Vitamin E and caffeic acid.

**Conclusion:**

The ethanolic extract of bark of *S. latifolius* showed potent antioxidant effects and low AChEI activity, high phenolic and flavonoid contents and presence of pharmacologically active compounds. These findings explain its wide usages in traditional medicine.

## Background

Due to its high reactivity, Oxygen is capable of becoming part of potentially damaging molecules called reactive oxygen species (ROS) [1]. Humans have evolved a highly sophisticated antioxidant protective system, both endogenous and exogenous in origin [2]. Whenever the balance between ROS production and antioxidant effect is disturbed, ‘oxidative stress’ results leading to various pathological conditions [3]. Many researchers have focused on antioxidant activity of phenolic compounds especially flavonoids and a positive correlation was observed [4–6]. ROS are playing a dangerous role in the pathogenesis of various diseases, including neurodegenerative disorders, cancer and artherosclerosis [7, 8]. Oxidative processes are the pathogens associated with the central nervous system in Alzheimer’s disease (AD). The brain in particular is highly vulnerable to oxidative damage as it consumes about 20% of the body’s total oxygen, with a high content of polyunsaturated fatty acids and lower levels of endogenous antioxidants [9, 10]. The brain of patients suffering from AD is said to be under oxidative stress. [11, 12]. AD is the most common neurodegenerative disorder, characterized clinically by progressive memory deficits and impaired cognitive function [13]. It is a major public health concern due to the increasing number of sufferers, placing strains on caregivers as well as on financial resources [14]. A deficiency in levels of the neurotransmitter acetylcholine (ACh) has been observed in the brains of AD patients, and inhibition of acetylcholinesterase (AChE), the key enzyme which hydrolyses ACh, is a major treatment option for AD [15]. Traditionally plants have been shown to be good options in the search for AChE inhibitors. Recently, several plants have been identified as containing AChEI activity [7]. In this respect, medicinal plants provide a rich source of biologically active constituents with multiple activities. *Sarcocephalus latifolius* Sm. (family: *Rubiaceae*), locally known as “Karmadoda”, have many uses in traditional medicine including malaria, dysentery, fever, hypertension and health promotion (antioxidant) [16–18].

## Methods

### Plant materials

Bark samples of *S. latifolius* were collected from South Kordfan state in February 2015. Identified and authenticated by Prof. Hatel H. Alkamali, Faculty of Science and Technology, Omdurman Islamic University, and confirmed by plant taxonomists at the herbarium of Medicinal and Aromatic Plants Research Institute, National Center for Research. Khartoum, Sudan.

### Extraction

The fresh samples were dried in shade for seven days, pulverized then used for extraction. Cold maceration methodology was carried out according to published method of Osama and Awdelkarim, 2015 [19].

### Fractionation

The crude extract was fractionated using liquid- liquid extraction methodology, which were carried by dissolving the samples in dist. H_2_O then partitioned between n-hexane, chloroform, ethyl acetate and n-butanol respectively using separating funnel apparatus.

### Qualitative phytochemical evaluation

Phytochemical screening was conducted to determine the presence of natural products in the fractions of selected plants using standard methods [20, 21].

### Determination of total phenolic content

**Fig. 1:**
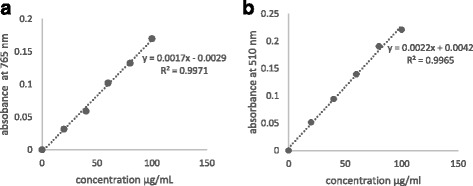
Standard curves of (**a**) Gallic acid and (**b**) Quercetin shows the absorbance against concentration

Total phenolic content was determined by Folin Ciocalteu method [22]. Calibration curve was constructed using gallic acid standards Fig. 1 and the total phenolic content was expressed as mg gallic acid equivalents (GAE)/g dry weight (DW).

### Determination of flavonoid content

The flavonoid content was measured by the aluminium chloride colorimetric assay [23]. Calibration curve was plotted using quercetin standards and flavonoid content was expressed as mg quercetin equivalents (QE)/g DW.

### GC-MS analysis

GC-MS analysis was carried out using GCMS instrument (Model GCMS-QP2010 Ultra, Shimadzu Co., Japan) equipped with a capillary column Rtx-5 (0.25 휇m film × 0.25 mm i.d. × 30 m length). The instrument was operated in electron impact mode at ionization voltage (70 eV), injector temperature (250 ^∘^C), and detector temperature (280 ^∘^C). The carrier gas used was helium (99.9% purity) at a flow rate of 1.2 mL/min and about one 휇L of the sample was injected. The oven temperature was initially programmed at 110 °C (7 min) to 200 °C at 10 °C/min and from 200 to 280 °C at 5 °C/min withhold time 0 and 9 min respectively. The identification of compounds from the spectral data was based on the available mass spectral records (NIST and WILEY libraries).

### Antioxidant activity: 2,2-diphenyl-1-picrylhydrazyl (DPPH) radical scavenging assay

The test samples were prepared in DMSO as 10× stocks from each test concentration (between 0 and 100 μg/ml) and briefly sonicated when necessary in an ultrasonic water bath. Solvents fractions producing radical scavenging activities equal to or higher than 50% at 100 μg/mL in a preliminary screen were further tested and IC_50_ (concentration of the sample producing 50% scavenging of DPPH radicals) determined using EZ-Fit Enzyme Kinetic Program. propyl gallate was tested in the assay as positive control. The assay method used in the present study was based on Shimada et al.*,* 1992 [24] method. The samples stock solutions (20 μL/well) were dispensed in triplicate onto 96-well plates. The assay was started with the addition of DPPH reagent (300 μM in ethanol, 180 μL/well). Appropriate blanks were prepared using the solvent only in addition to the same amount of DPPH reagent to get rid of any inherent solvent activity. Negative controls were also run in parallel to correct for any non-DPPH absorbance by coloured extracts at the test wavelength. The plate was immediately shaken for 30 s and incubated in the dark for 30 min at room temperature. The remaining DPPH was measured in the microplate reader at 517 nm. Percentage of radical scavenging activity (RSA) was calculated as following:

$$ \%\mathrm{RSA}=100-\left\{\left(\mathrm{Ac}-\mathrm{At}\right)/\mathrm{Ac}\right\}\times 100 $$

Where, At = Absorbance value of test compound; Ac = Absorbance value of control.

### The multi-well plate AChE inhibition assay

The AChE inhibitory activity was tested using 96 well micro-plate assay based on Ellmam et al.*,* 1961 [25] method with minor modifications. Each extract (10 μL of 5 mg/mL in ethanol) was dispensed in triplicate onto 96 well microplate and mixed with 190 μL of Ellman’s mixture containing 20 μL of enzyme, 140 μL to phosphate buffer, pH 8, containing 10 μL of 0.5 mM of 5, 5’- dithio-bis-(2-nitrobenzoic acid) (DTNB, Sigma-Aldrich, Germany) and 20 μL acetylthiocholine iodide (ATCI, Sigma-Aldrich, Germany). The control wells contained ethanol instead of the extract. The enzymatic activity was monitored at 412 nm every 30 s intervals for 3 min (linear reaction). The enzyme rate was calculated from the slope of the curve of absorbance vs time. As screening strategy, final concentration of 1000 μg/mL from each extract was examined and the average % inhibition was calculated relative to the enzyme rate at the control wells according to the following equation:

$$ \%\mathrm{Inhibition}=100-\left\{\left(\mathrm{Ac}-\mathrm{At}\right)/\mathrm{Ac}\right\}\times 100 $$

### MTT (3-(4, 5-dimethylthazol-2-yl)-2, 5-diphenyl tetrazonium bromide) cytotoxicity assay

In the present investigation, Vero (normal, African green monkey kidney) cell line was used and cytotoxicity on these cells was assessed as described previously [26]. For each experiment, cultures were seeded from frozen stocks. Vero cells were maintained complete medium consisting of 10% fetal bovine serum and 90% minimal essential medium (MEM). The cells were incubated at 37 °C in a 5% CO_2_ atmosphere and were in the logarithmic phase of growth at the time of the neutral red (NR) and tetrazolium (MTT) assays. Cells were harvested and seeded into 96-well tissue culture plates at a density of 1 × 10^4^ cells per well of aliquots of medium (200 μL). The cells were allowed to adhere to the wells for 24 h at 37 °C in a humid atmosphere optimized with 5% CO_2_ in air. The next day, the plant fractions were added at the desired final concentrations and incubated for 72 h. All experiments were performed at least four times. Phosphate-buffered saline (PBS) was used as a negative. After the 72 h exposure period, the toxic endpoints were determined at 570 nm. Viability was defined as the ratio (expressed as a percentage) of absorbance of treated cells to untreated cells that served as negative control.

### Statistical analysis

All data were expressed as means ± SD for three experiments. *P* values <0.05 were considered statistically significant. Statistical analyses were performed using Excel software (Microsoft 2010).

## Results and discussion

### Extraction yield and phytochemical screening

The percentage yield of *S. latifolius* ethanolic extract was 47.33% of dry weight. The polar fractions (water and butanol) showed the highest percentage yield (Table 2), this could be attributed to the polar nature of the crude extract obtained with polar solvent (ethanol).

The results of preliminary investigation on the phytochemicals present in different solvent fractions are presented in Table 1. Different phytoconstituents such as phenolics, flavonoids, tannins, alkaloids, saponins, quinones, steroids, and terpenoids were detected in the tested fractions. The phytochemicals investigated in the present study are known to be beneficial in industrial and medicinal sciences [27]. Also, this preliminary knowledge can be looked at as a decipher in the search of a new source of economically valued chemical compounds [28, 29].

**Table 1:** Preliminary screening of secondary metabolites in the fractions of *S. latifolius*

Family of compound	Type of test	interference				
		*n*-hexane	CHCl_3_	EtOAc	n-BuOH	H_2_O
Phenols	FeCl_3_	+v	+v	+v	+v	+v
Tannins	FeCl_3_	-v	+v	+v	+v	+v
Flavonoids	KOH	+v	+v	+v	+v	+v
	Alkaline	+v	+v	+v	+v	+v
	Lead acetate	+v	+v	+v	+v	+v
Quinones	H_2_SO_4_	-v	+v	+v	+v	+v
Alkaloids	Dragendorff’s	+v	+v	+v	+v	+v
	Wagner’s	+v	+v	+v	+v	+v
Triterpenes	Salkowski	+v	+v	+v	+v	+v
Diterpenes	Copper acetate	-v	-v	-v	-v	-v
Steroids	Salkowski	+v	+v	+v	+v	+v
Saponins	Forth	-v	+v	+v	-v	+v

+ve positive -ve negative

### Total phenolic and flavonoid contents

**Fig. 2:**
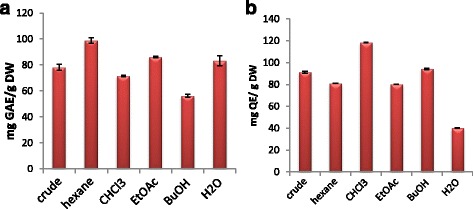
Comparison charts of (**a**) Total phenolic content. **b** Total flavonoid content of ethanolic extract and solvent fractions of *S. latifolius*

Crude natural extracts and compounds purified from these extracts can serve as better herbal drug sources owing to their fewer side effects and nutritional value [30]. As presented in Table 2, the ethanolic crude extract of *S. latifolius* bark exhibited total phenolic content of 78.21 ± 2.4 mg GAE/g DW. Fig. 2 a similar study carried out using the methanolic leaf and root extracts of *S. latifolius*, has shown that the total phenolic content present was (0.016 ± 0.03 and 0.036 ± 0.05 mg GAE/g DW) respectively, which is very low compared to the present study [31]. This high variation could be due to many reasons including, the part of plant under study, which contains different chemical composition with different percentage, the method, solvent used for extraction, the origin of plant samples, and the environmental factors. The hexane fraction showed the highest phenolic content (98.78 ± 2.1 mg GAE/g DW), this result indicates the presence of high lipid soluble phenolic compounds such as vitamin E, whose existence was confirmed by GCMS analysis.

**Table 2:** Yield percentages, Total phenolic and flavonoid contents of ethanolic extract and solvent fractions of *S. latifolius*

Sample	Extraction yield (*w*/w% of dry weight)	Phenolic content (mg /g GAE)	Total flavonoid (mg/g QE)
Crude	47.33	78.21 ± 2.4	91.36 ± 0.84
Hexane	6.18	98.78 ± 2.1	81.01 ± 0.012
CHCl_3_	1.64	71.49 ± 0.5	118.29 ± 0.21
EtOAc	3.09	86.12 ± 0.7	80.23 ± 0.03
BuOH	19.09	56.20 ± 1.23	94.32 ± 0.71
H_2_O	70.91	83.20 ± 3.7	40.22 ± 0.28

Successful determination of biologically active compounds from plant material is largely dependent on the type of solvent used in the extraction procedure. Higher concentrations of more bioactive flavonoid compounds were detected with 80% ethanol [32]. Therefore, ethanol was chosen for extraction. Plant phenolic compounds especially flavonoids are currently receiving greater interest due to their antioxidants potential [27, 33]. Aluminium chloride colorimetric assay yielded total flavonoid content of (91.36 ± 0.84 mg QE/g DW) for the crude extract. Highest flavonoid contents were observed in chloroform fraction (118.29 ± 0.21 mg QE/g DW), which indicate the high amount of less polar flavonoids (aglycones) such as isoflavones, flavanones, highly methoxylated flavones, and flavonols [34]. These results could give a clue interpreting the observed high bioactivities of this plant.

### Antioxidant and AChE inhibitory activities

**Fig. 3:**
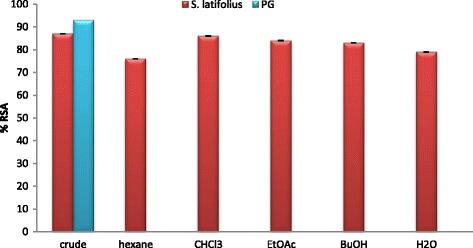
Percentage of RSA of *S. latifolius* ehanolic extract and solvent fractions

**Fig. 4:**
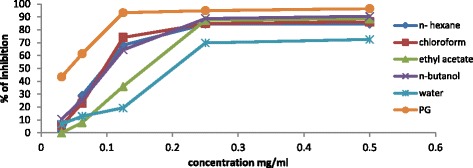
Comparison Chart of Antioxidant potentials of *S. latifolius* fractions with the decrease of concentration (IC50 evaluation)

Recently, interest has increased in naturally occurring antioxidants that can be used to protect human beings from oxidative stress damages [35, 36]. In the current study, the ethanolic crude extract exhibited high antioxidant activity with 87 ± 0.03%. The order of the activity (IC_50_ mg/ml) was as follow: hexane (0.098 ± 0.08) > chloroform (0.099 ± 0.029) > butanol (0.104 ± 0.19) > ethyl acetate (0.148 ± 0.33) and eventually water fraction (2.015 ± 0.3), Table 3 and Figs. 3, and 4 illustrate these results. The activity of hexane and chloroform fractions (0.098 ± 0.08 and 0.099 ± 0.029 mg/ml) respectively, is comparable values to the standard antioxidant PG (0.0414 ± 0.11 mg/ml); they also showed high amount of phenolic and flavonoid contents, it’s possible that could be the reason of this high antioxidant potential.

**Table 3:** Antioxidant activity (%RSA and IC_50_) of solvent fractions of *S. latifolius*

Sample	% RSA	IC_50_ (mg/ml)
Hexane	76 ± 0.02	0.098 ± 0.08
CHCl_3_	86 ± 0.03	0.099 ± 0.029
EtOAc	84 ± 0.03	0.148 ± 0.33
BuOH	83 ± 0.02	0.104 ± 0.19
H_2_O	79 ± 0.10	2.015 ± 0.3

Inhibition of AChE has been considered as a promising strategy for the treatment of neurological disorders such as Alzheimer’s disease, senile dementia, ataxia and myasthenia gravis, in which a deficit in cholinergic neurotransmission is involved [37, 38]. The side effects of anti-AChE drugs such as toxicity, tolerability, and loss of efficiency stimulates the researchers to screen alternative natural anti-AD drugs for medication switch [39]. Ethanolic extract displayed low AChE inhibitory activity with (40.2 ± 0.10). GCMS analysis of chloroform fraction indicates the presence of caffeic acid which has been reported to be a potent inhibitor of both AChE and BChE [40]. It is possible that the solvent used for extraction was not able to isolate the active ingredients with a proper amount.

### GCMS analysis

**Fig. 5:**
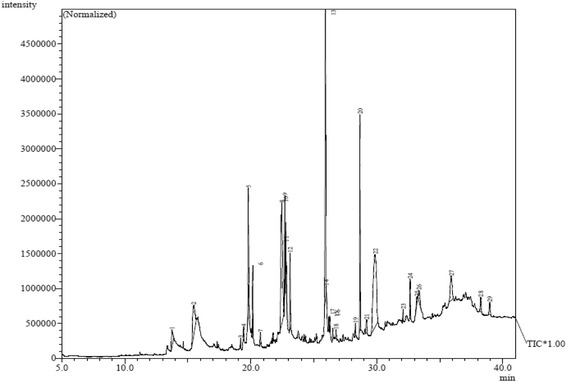
GC-MS chromatogram for hexane fraction of *S. latifolius* bark

**Fig. 6:**
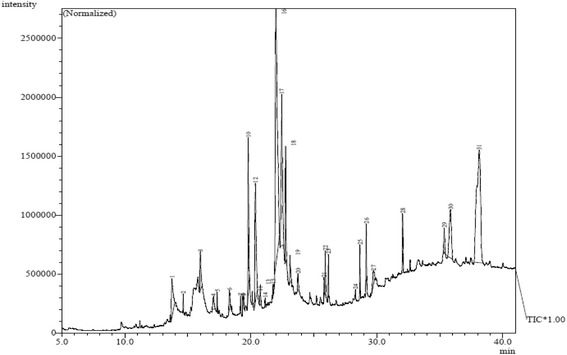
GC-MS chromatogram for chloroform fraction of *S. latifolius* bark

Due to their superior antioxidant activities hexane and chloroform fractions were analyzed with GC-MS, to identify their chemical composition which may be responsible of the measured activities. The GC-MS analysis lead to the identification of a number of compounds. These compounds were identified through mass spectrometry attached with GC. Interpretation of mass spectrum was conducted using the database of National Institute Standard and Technology (NIST). The name, molecular weight and structure of the components of the test materials were ascertained, illustrated in Tables 4, and 5 and Figs. 5, and 6.

**Table 4:** GC-MS spectral analysis of hexane fraction of *S. latifolius* bark

Peak no.	R. Time	Area %	Compunde name	Molecular Formula	Mass
1	13.736	1.68	5,5-Dimethyl-1,5-oxasilonan-9-one	C_9_H_18_O_2_Si	186
2	15.467	5.67	Quinic acid	C_7_ H_12_ O_6_	192
3	19.188	0.29	Pentadecanoic acid, methyl ester	C_16_ H_32_ O_2_	256
4	19.425	0.67	Ethyl (2E)-3-(4-hydroxy-3-methoxyphenyl)-2-propenoate	C_12_H_14_O_4_	222
5	19.805	6.89	Pentadecanoic acid	C_15_H_30_O_2_	242
6	20.147	2.20	Palmitic acid ethyl ester	C_18_ H_36_ O_2_	284
7	20.761	0.53	2-Cyclopropylideneadamantane	C_13_H_18_	174
8	22.455	16.49	Oleic Acid	C_18_H_34_O_2_	282
9	22.694	3.23	Methyl linoleate	C_19_ H_34_ O_2_	294
10	22.765	1.64	Ethyl octadec-9-enoate	C_20_H_38_O_2_	310
11	22.814	0.48	Linolenic acid methyl ester	C_19_ H_32_ O_2_	292
12	23.122	3.91	Heptadecanoic acid, ethyl ester	C_19_ H_38_ O_2_	298
13	25.920	14.21	Oelic acid amide	C_18_ H_35_ N O	281
14	25.994	0.23	Oelic acid amide	C_18_ H_35_ N O	281
15	26.187	0.44	Palmitic acid ethyl ester	C_18_ H_36_ O_2_	284
16	26.265	0.60	Stearic acid amide	C_18_H_37_NO	283
17	26.509	0.43	2-Pentyl-2-nonenal	C_14_H_26_O	210
18	26.761	0.43	Oxirane, hexadecyl-	C_18_H_36_O	268
19	28.299	0.48	Octadecanal	C_18_H_36_O	268
20	28.665	9.23	Phthalic acid, mono-(2-ethylhexyl) ester	C_16_H_22_O_4_	278
21	29.192	0.97	Ethyl palmitate	C_18_ H_36_ O_2_	284
22	29.881	19.50	Cinchol	C_29_ H_50_ O	414
23	32.086	0.57	Ethyl docosanoate	C_24_H_48_O_2_	368
24	32.642	1.64	Spinacene	C_30_H_50_	410
25	33.176	0.59	3-[(Trimethylsilyl)oxy]lanosta-8,24-diene	C_33_H_58_OSi	498
26	33.343	1.44	Lupenyl acetate	C_32_H_52_O_2_	468
27	35.907	3.46	Stigmast-4-en-3-one	C_29_H_48_O	412
28	38.248	1.09	Cholesteryl bromide	C_27_ H_45_ Br	448
29	38.974	1.01	Vitamin E	C_29_H_50_O_2_	430

**Table 5:** GC-MS spectral analysis of chloroform fraction of *S. latifolius* bark

Number	R. Time	Area %	Compound name	Molecular Formula	Mass
1	13.735	2.97	5,5-Dimethyl-1-oxa-5-silacyclononanone-9	C_9_H_18_O_2_Si	186
2	14.635	0.29	9-Eicosene, (E)-	C_20_H_40_	280
3	15.989	1.99	2H–Pyran-2-one, 5-ethylidenetetrahydro-4-(2-hydroxyethyl)-	C_9_H_14_O_3_	170
4	17.029	0.85	4-((1E)-3-Hydroxy-1-propenyl)-2-methoxyphenol	C_10_H_12_O_3_	180
5	17.334	0.35	1-Heptadecene	C_17_H_34_	238
6	18.302	0.84	p-Hydroxycinnamic acid, ethyl ester	C_11_H_12_O_3_	192
7	19.187	0.35	9-Octadecenoic acid, 12-(acetyloxy)-, methyl ester, [R-(Z)]-	C_21_H_38_O_4_	354
8	19.361	0.18	7,9-Di-tert-butyl-1-oxaspiro(4,5)deca-6,9-diene-2,8-dione	C_17_H_24_O_3_	276
9	19.422	0.15	alpha.-D-Xylofuranose, 1,2-O-isopropylidene-5-(t-butyldimethylsilyl)-	C_14_H_28_O_5_Si	304
10	19.800	5.89	Pentadecanoic acid	C_15_ H_30_ O_2_	242
11	20.115	0.30	9-Tricosene, (Z)-	C_23_H_46_	322
12	20.360	7.84	2H-1-Benzopyran-2-one, 7-hydroxy-6-methoxy-	C_10_ H_8_ O_4_	192
13	20.761	0.62	Bicylo[4.1.0]heptane, 7-bicyclo[4.1.0]hept-7-ylidene-	C_14_H_20_	188
14	21.139	0.22	Trimethylsilyl 3-methoxy-4-(trimethylsilyloxy)cinnamate	C_16_H_26_O_4_Si_2_	338
15	21.769	0.31	9-Octadecenoic acid (Z)-, methyl ester	C_19_H_36_O_2_	296
16	21.996	25.46	2-Propenoic acid, 3-(3,4-dihydroxyphenyl)-	C_9_ H_8_ O_4_	180
17	22.450	9.78	Oleic Acid	C_18_H_34_O_2_	282
18	22.762	3.28	Octadecanoic acid	C_18_H_36_O_2_	284
19	23.104	1.19	1-Hexadecene	C_16_ H_32_	224
20	23.740	0.61	9,12-Octadecadienoic acid (Z,Z)-	C_18_H_32_O_2_	280
21	25.816	0.39	O O′-BIPHENOL, 4,4’,6,6’-TETRA-T-BUTYL-	C_28_ H_42_ O_2_	410
22	25.909	1.08	9-Octadecenamide, (Z)-	C_18_ H_35_ N O	281
23	26.155	0.93	1-Nonadecene	C_19_H_38_	266
24	28.301	0.19	Oxirane, heptadecyl-	C_19_H_38_O	282
25	28.662	1.17	1,2-Benzenedicarboxylic acid, mono(2-ethylhexyl) ester	C_16_H_22_O_4_	278
26	29.163	1.28	1-Nonadecene	C_19_H_38_	266
27	29.732	1.15	gamma.-Sitosterol	C_29_H_50_O	414
28	32.053	1.83	1-Triacontanol	C_24_H_50_O	354
29	35.350	1.02	17-Pentatriacontene	C_35_H_70_	490
30	35.848	5.09	Methyl commate C	C_31_ H_50_ O_4_	486
31	38.123	22.40	Lup-20 (29)-en-3-ol, acetate, (3.beta.)-	C_32_H_52_O_2_	468

GC-MS spectrum of the hexane and chloroform fractions revealed the presence of 29 and 31 compounds respectively. Two phenolic compounds (Ethyl (2E)-3-(4-hydroxy-3-methoxyphenyl)-2-propenoate and Vitamin E) were observed in hexane fraction. Fat soluble vitamin E is one of the most active natural antioxidants, it is the most effective chain-breaking antioxidant within the cell membrane where it protects membrane fatty acids from lipid peroxidation. The supplemental intakes of this powerful antioxidant have been documented to be useful against cancer [41]. Vitamin E also acts in the prevention of free radical formation.

chloroform fraction declared the presence of five phenolic substances (4-((1E)-3-Hydroxy-1-propenyl)-2-methoxyphenol; p-Hydroxycinnamic acid, ethyl ester; 2H-1-Benzopyran-2-one, 7-hydroxy-6-methoxy; Caffeic acid, O O’-biphenol, 4,4’,6,6’-tetra-T-butyl) these phenolic compounds are known antioxidants, therefore the antioxidant potentials of this plant could be justified. However, further studies on the isolation, characterization, and biological evaluation of these identified compounds are necessary to confirm their potential benefits.

### Cytotoxicity study

MTT assay reveled that, all tested fractions have no toxic effects on Vero cells with IC_50_ more than 200 μg/ml, these results are shown in (Table 6).

**Table 6:** The cytotoxic effect (expressed as % inhibition and IC50 values) of hexane and chloroform fractions of *S. latifolius* tested at 125, 250 and 500 μg/ml against Vero cells

Code of extract	Concentration (μg/ml)			IC_50_ (μg/ml)	IC_50_
	Inhibition % ± SD				
	500	250	125		
Hexane	55.3 ± 0.04	50.4 ± 0.02	40.9 ± 0.03	277.8	>100
Chloroform	67.6 ± 0.08	55.0 ± 0.01	35.9 ± 0.05	224.9	>100
Control	95.3 ± 0.00				<30

IC_50_ < 30 μg/ml: High toxic. Control = triton was used as positive control at 0.2 μg/ml. the maximum concentration used was 500 μg/ml

The MTT assay is a test of metabolic competence based upon assessment of mitochondrial performance relying on the conversion of yellow MTT to the purple formazan derivative by mitochondrial succinate dehydrogenase in viable cells [42]. Increasing concentrations of the tested fractions did not affect mitochondrial respiration as measured by the MTT cytotoxicity assay. However, the results of this assay measuring cell integrity showed that these solvent fractions are not toxic over this concentration range tested.

## Conclusion

*S. latifolius* is a Sudanese medicinal plants commonly used as herbal medicine for several purposes. In the present study, the selected plant was investigated in vitro for AChEI and antioxidant properties. In addition, the total phenolic and flavonoids contents were measured. GC-MS analysis of *S. latifolius* (hexane and chloroform) fractions reveled the presence of well known antioxidant compound such as Vitamin E and Caffeic acid. According to these findings it could be suggested that *S. latifolius* (hexane and chloroform) fractions might be potent and safe antioxidant materials in medicine or food industry.

## Abbreviations

Abs: Absorbance
Ac: Absorbance value of Control
Ach: Acetylcholine
AChE: Acetylcholinesterase
AChEI: Acetylcholinesterase Inhibitory
AD: Alzheimer’s Disease
At: Absorbance value of Test compound
BuOH: Butanol
DMSO: Dimethyl sulfoxide
DPPH: 2,2-diphenyl-1-picryl hydrazyl
DTNB: 5,5’-Dithiobs (2-nitro benzoic acid)
EI: Electron Ionization
EtOAc: Ethyl Acetate
FBS: Fetal Bovine Serum
GAE: Gallic Acid Equivalent
GC: Gas Chromatography
GC-MS: Gas Chromatography- Mass Spectrometer
MEM: Minimal Essential Medium
MTT: Microculture tetrazolium
NIST: National Institute of Standards and Technology
PG: Propyl Gallate
QE: Quercetin Equivalent
RSA: Radical Scavenging Activity
